# Treatment planning for metals using an extended CT number scale

**DOI:** 10.1120/jacmp.v17i6.6153

**Published:** 2016-11-08

**Authors:** John P. Mullins, Michael P. Grams, Michael G. Herman, Debra H. Brinkmann, John A. Antolak

**Affiliations:** ^1^ Department of Radiation Oncology Mayo Clinic Rochester MN USA; ^2^ Department of Radiation Oncology Methodist Estabrook Cancer Center Omaha NE USA

**Keywords:** extended CT scale, metal implant dosimetry

## Abstract

Metal implants which saturate the CT number scale may require dosimetrist and physicist involvement to manually contour and assign an appropriate value to the metal for accurate dose calculation. This study investigated dose calculation based directly on extended CT scale images for different metals and geometries. The aim was to evaluate extended CT accuracy as a suitable alternative to standard CT methods in the presence of high‐Z materials and artifacts, despite the reduced HU resolution of extended CT. Gafchromic film measurements were made for comparison to calculated doses. The method of direct dose calculation on extended CT scale was compared to our institution's standard method of manually contouring and assigning metal values on saturated CT images for each of the metal samples. Clinical patient plans with metal implants were investigated and DVHs were compared between standard CT and extended CT dose calculations. Dose calculations showed agreement within 2% between the two methods of metal characterization and the film measurement in the case of the strongest metal attenuator, cobalt‐chromium. In the clinical treatment plans, the greatest dose discrepancy between the two methods was 1.2%. This study suggests that direct dose calculation on an extended scale CT image in the presence of metal implants can produce accurate clinically viable treatment plans, thereby improving efficiency of clinical workflow and eliminating a potential source of human error by manual CT number assignment.

PACS number(s): 87.55.dk

## I. INTRODUCTION

Metals are routinely encountered in radiotherapy patients in the form of orthopedic implants, dental fillings, and subcutaneous medical devices. Metals can cause image artifacts and saturate the CT number scale due to high atomic number and density. Treatment planning in the presence of metals can be handled by contouring the high‐density material and scatter artifacts, and manually assigning a CT number to those regions corresponding to the desired relative electron density (RED).^(1)^ Metal artifact reduction (MAR) algorithms have been studied with respect to image improvement and treatment planning accuracy.^2^, ^3^, ^4^, ^5^, ^6^, ^7^ Regardless of how scatter artifacts are handled, the CT number of the metal itself must accurately correspond to a RED in the treatment planning system (TPS) for proper handling of dose calculation.

**Figure 1:**
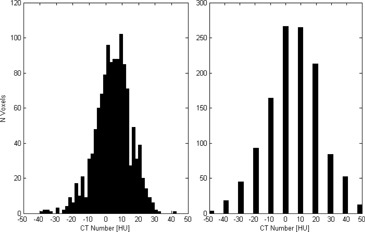
For the same ROI, the histogram standard CT scale image has voxel value resolution of 1 HU (left). The extended CT scale image (right) has the same mean and deviation, but a voxel value resolution of 10 HU.

The CT scale saturates at 3071 Hounsfield units (HU) on typical 12‐bit images, reflecting a DICOM slope of 1 HU and an intercept of −1024 HU applied to 4096 available unsigned integers of bit‐depth in the DICOM file. Glide‐Hurst et al.^(8)^ have investigated the accuracy of extended CT scale with and without MAR on a Phillips scanner which implements extended scale by acquiring full 16 bit‐depth data. Our institution uses Siemens scanners (Somatom Definition AS, Siemens, Munich, Germany), which implement extended CT scale by storing 12‐bit data and altering the slope and intercept in the DICOM header to a slope of 10 and intercept of −10240 HU, thereby expanding the HU value range tenfold and increasing voxel value granularity to 10 HU (Fig. 1). On the Siemens software version (ver. VA_46) installed at our institution during this study, extended CT scale and MAR options were mutually exclusive; therefore, extended scale without MAR was investigated.

Our institution's current standard of practice involves the dosimetrist contouring scatter artifacts to be manually assigned a CT number corresponding to the RED of water or surrounding tissue. The metal is contoured and a physicist is consulted to provide a CT number corresponding to the RED of the metal present. Common medical implant materials are stainless steel (SS), titanium (Ti), and cobalt chromium (CoCr). These all saturate the standard CT scale at 3071 HU. The process to determine the composition of the metal may involve searching surgical notes for a manufacturer and model of the device and researching device‐specific compositions. This process is time‐consuming and could introduce systematic uncertainty if generic CT number values chosen for the metals differ from the implant‐specific alloys. Similarly, the act of contouring the metal implant is time‐consuming and could introduce systematic uncertainty if the size of the implant is not contoured accurately. Metal contouring is made more difficult by CT dilation artifacts.^9^, ^10^

If extended CT was proven suitable for direct use in dose calculation, this method could decrease treatment planning time and remove a step of human intervention that could potentially introduce error. This study investigates dosimetric accuracy for dose calculations in the presence of metals for the Siemens implementation of extended CT. Additionally, this study presents some representative clinical cases to assess the relative impact of calculating a dose distribution based on manual CT number assignment versus direct calculation on extended CT scans.

## II. MATERIALS AND METHODS

**Figure 2:**
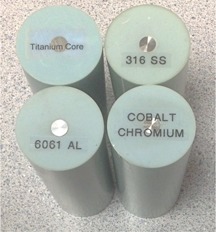
Custom metal sample plugs designed from plastic‐water inserts for the CIRS Model 062 RED phantom. CT number values from these samples were used as data points to characterize the extended CT scale HU to RED conversion curve in the extended range.

The study consisted of two components: a phantom study using film measurements to quantify the accuracy of extended CT, and a retrospective patient study to compare dosimetric impact between extended and standard CT methods. For dose calculations performed on extended CT scale, a lookup table converting CT number to RED was created based on a commercial RED phantom (CIRS Model 62, Computer Imaging Reference Systems, Norfolk, VA). Additional custom plugs were machined to contain metal samples of 6061 alloy aluminum (Al), Ti, 316 alloy SS, and 6B alloy CoCr (High Performance Alloys, Tipton, IN), (Fig. 2). The HU conversion table was stored in the TPS for dose calculations performed using extended CT.

### A. Phantom study

**Figure 3:**
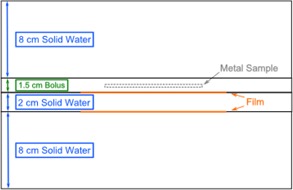
Phantom setup with relative positions of metal and film.

The phantom study was performed to measure dosimetric agreement of extended and standard CT methods using film measurements for a variety of metal types and geometries. Metal samples included cylindrical rods (6 mm diameter of Al, steel and CoCr), sheets (2 mm thick of steel, SS, and copper (Cu)), and brass mesh. Brass mesh was of interest due to potential clinical use as a bolus material.^(11)^ Each metal sample was placed individually between layers of Superflab bolus (Mick Radio‐Nuclear Instruments, Mount Vernon, NY). Phantoms were constructed by placing 1.5 cm bolus containing a metal sample between solid water blocks of 8 cm and 10 cm thickness (Fig. 3). EBT3 Gafchromic film (Ashland, Bridgewater, NJ) was placed between phantom layers in two locations, one 0.5 cm posterior to the metal sample and another 2.5 cm posterior.

Phantoms were CT simulated and treatment plans were created in the Eclipse TPS (ver. 13.6, Varian Medical Systems, Palo Alto, CA). Two distinct planning methods were performed, referred to subsequently as “standard CT” and “extended CT.” For the standard CT method, metal and scatter artifacts were contoured on a standard scale CT and the metal contour was manually assigned a CT number corresponding to the RED of the metal present. Scatter artifacts in the surrounding tissue were assigned the CT number of water. For the extended CT method, an extended CT scale was used and scatter artifacts were contoured and assigned a CT number of water. The metal was not contoured or manually assigned a CT number, but was converted to RED by the extended scale HU to RED conversion curve in the TPS. These methods were also compared to a TPS calculation on a standard HU scale CT where no metal contouring or HU override interventions were performed (referred to as “control”).

Contouring of metal and artifacts was performed with the assistance of an experienced dosimetrist to ensure standard practice was followed. Identical scatter contours were used for both the standard CT and extended CT methods. Dose was calculated with the anisotropic analytic algorithm (AAA version 11.0.31). Phantoms were irradiated with AP/PA parallel‐opposed $10\times 10\;{\text{cm}}^{2}$ fields, 135 MU each field, for a total dose to the center of the phantom of 215‐230 cGy, depending on the metal present. AP/PA fields were chosen (versus a single AP field) to reduce impact of vertical setup uncertainty on film measurements, as well as to better reflect impact on clinically relevant beam arrangements.

All film analysis was performed with FilmQA Pro software (Ashland, Bridgewater, NJ) using a one‐scan protocol and triple‐channel dosimetry.^12^, ^13^ Films were scanned in red‐green‐blue (RGB) format using a 48‐bit scanner (Epson Expression 10000 XL, US Epson, Long Beach, CA) at 72 dpi, in transmission mode, and with no color or sharpness corrections. Based on our commissioning of the film protocol used in this study, film measurements are reproducible to within 1% and agree with ion chamber measurements to within 1.5%.

Gamma analysis was performed for a fixed region of interest (ROI) comparing the film measurement to dose planes from the TPS for control, standard CT, and extended CT methods. Gamma criteria used were 3% agreement threshold to global maximum within 1 mm distance, excluding points below 10% of maximum dose. Percent of ROI pixels passing the criteria were tabulated.

### B. Retrospective patient study

Dose distributions were calculated with standard CT and extended CT methods for four different retrospective clinical treatment plans involving metal implants in various anatomical regions (two hip prostheses, a breast expander, and a femoral rod). Dosimetric differences were assessed by dose‐volume histogram (DVH) comparison. DVHs for target and normal structures of interest were overlaid for inspection of agreement.

## III. RESULTS & DISCUSSION

### A. Phantom study

**Figure 4:**
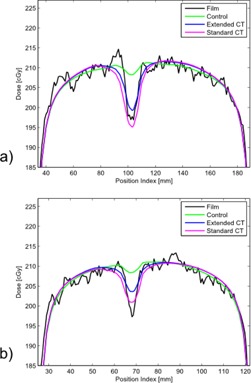
Dose profiles 0.5 cm posterior (a) and 2.5 cm posterior (b) to CoCr rod sample.

**Figure 5:**
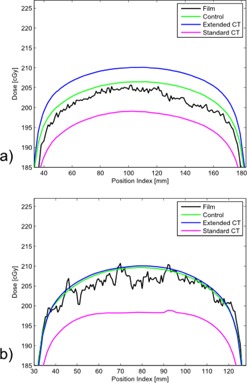
Dose profiles 0.5 cm posterior to (a) SS sheet and (b) carbon steel sheet.

**Figure 6:**
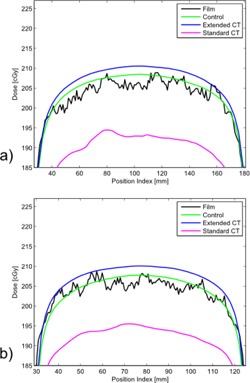
Dose profiles 0.5 cm posterior (a) and 2.5 cm posterior (b) to brass mesh.

Dose profiles were compared between control, standard CT, and extended CT methods versus the measured dose to film. Notable representative examples are shown in Figs. 4–6. Dose profiles for the CoCr rod, the strongest attenuator investigated, indicated TPS dose calculations distal to the CoCr rod agree within 2% between the standard and extended CT methods (Fig. 4).

Dose profiles between the steel and SS alloy sheets show little difference between the two metal types (Fig. 5). The similar agreement between the extended scale profile and film measurement for each metal sample indicates that subtle material composition differences do not result in noticeable changes to extended CT scale HU value or dose calculation. The standard CT dose calculation for each sample resulted in an underestimation of the dose delivered posterior to the metal by almost 6%. The apparent sizes of the metal samples on CT were dilated due to artifact and, although the dosimetrist attempted to account for the effect in contouring, the manually defined regions were still larger than true attenuator thickness. The extent of overcontouring was consistent between the two independent plans. This demonstrates the potential for introduction of systematic uncertainty when manually contouring metals.

**Figure 7:**
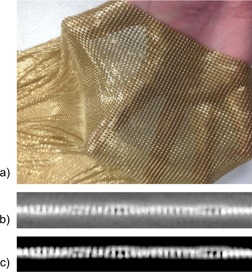
Brass mesh bolus (a) visual appearance, (b) CT appearance with W/L=2450/1025 HU, (c) CT appearance with W/L=1450/1375 HU.

Systematic uncertainty resulting from contouring had the greatest impact for the brass mesh sample (Fig. 6). The dose calculation from standard CT resulted in underestimation of dose by 6%‐7%. Dilation artifact throughout the fine structure of the mesh resulted in a thicker and more solid appearance on CT, even with window/level settings applied intending to mitigate the expected presence of dilation artifact. The physical appearance of the brass mesh and its appearance on CT at two different window/level settings are shown in Fig. 7. Extended versus standard scale CT images did not result in a visible difference of the mesh thickness. However, partial volume averaging of the metal attenuator through the voxels surrounding the metal sample on the extended scale image resulted in satisfactory dose calculation by the TPS (within ∼2% dose compared to film) with no manual intervention other than contouring obvious scatter artifacts.

The magenta lines in Figs. 5 and 6 represent the result of our institution's current clinical standard of practice in the presence of challenging metals. The standard CT method is capable of giving an accurate result, but the method depends on accurate manual inputs. The metal contour (obfuscated by CT artifacts) and choice of CT number (dependent on knowledge of metal type present) must be manually determined. As evidenced by comparing the standard CT to the control (green lines) in Figs. 5 and 6, sometimes manual intervention can introduce additional error. In these two examples, the control performed as well as, or slightly better than, standard or extended CT. This may be due to metal dilation artifact conflicting with actual thin metal size used. The CoCr rod (Fig. 4) showed standard and extended methods to be a better agreement to measurement. The blue lines in Figs. 5 and 6 represent the result of a proposed alternative method based on extended CT that removes subjective manual steps from the treatment planning workflow. Time savings for extended CT have been estimated as 20–30 min for simple cases and 1 hr for complex cases. These time estimates are for metal and scatter contouring and do not include physics consultation for metal assignment.

Table 1 reports the gamma analysis results of TPS calculations compared to film measurements. Results indicate the standard CT method introduced human error in contouring and/ or HU assignment for the brass mesh and sheet metal cases. TPS calculations tend to be more accurate further distal to the metal sample.

**Table 1:** Gamma analysis of film measurements compared to TPS dose calculations. Gamma criteria are 3% threshold within 1 mm distance

	*Pixel Pass Rate(%)*		
*Material*	*Control*	*Standard*	*Extended*
*Film Position Relative to Metal Sample 0.5 cm Pasterior*			
Al Rod	97.7	97.7	99.9
Brass Mesh	99.9	0.2	97.6
CoCr Rod	95.2	99.4	100
Cu Sheet	78.2	55.9	95.8
Superflab (no metal)	100	100	100
SS Sheet	100	59.8	99.9
Steel Rod	94.2	99.8	99.6
Steel Sheet	99.9	46.5	99.6
*2.5 cm Posterior*			
Al Rod	100	100	99.8
Brass Mesh	99.3	0.6	99.1
CoCr Rod	96.3	100	99.8
Cu Sheet	100	69.3	100
Superflab (no metal)	98.9	98.9	99.4
SS Sheet	98.6	39.0	99.9
Steel Rod	94.9	99.9	99.4
Steel Sheet	99.3	49.9	100

### B. Retrospective patient study

**Figure 8:**
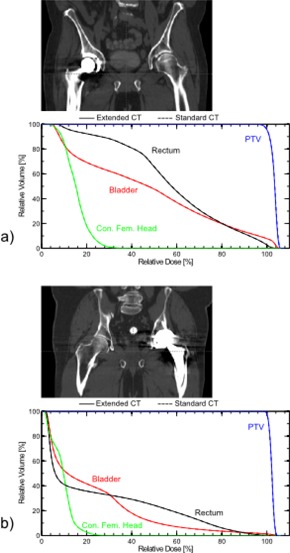
Anatomies and corresponding DVHs for VMAT plans on two different patients with hip prostheses containing portions of Ti, SS and CoCr: (a) prosthetic on patient's right hip and (b) on patient's left.

**Figure 9:**
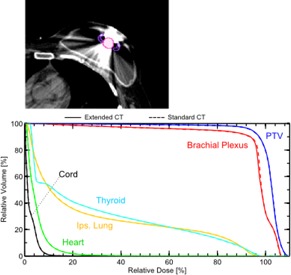
Anatomy and corresponding DVH for a breast tangent plan in the presence of an implant expander containing neodymium and Ti.

**Figure 10:**
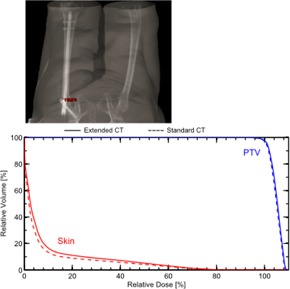
Anatomy and corresponding DVH for a three‐field, 3D‐conformal extremity plan in the presence of a Ti femur rod.

For the two clinical prostate plans involving hip implants (containing portions of Ti, SS, and CoCr), comparison between DVHs for plans using extended CT versus standard CT indicated no apparent differences. Lines for major structures appear superposed (Fig. 8). The breast tangent plan with an implant expander (neodymium and Ti) showed a dose discrepancy of about 0.5% in the brachial plexus at the point of maximal DVH discrepancy (Fig. 9). The three‐field, 3D‐conformal extremity plan containing a Ti femoral rod showed a maximal skin dose discrepancy of about 1.2% (Fig. 10). Without *in vivo* dosimetry, we do not know which of the calculation methods is more accurate in these examples, but the DVHs demonstrate that the two calculations are in close agreement. These examples suggest that the extended CT method could be substituted for the standard CT method, with little impact on calculated plan dose. As suggested by the phantom studies, the extended CT method could possibly be more accurate by removing a potential for introduction of human error. As well, extended CT may be more efficient by removing the manual contouring and assignment step from the planning workflow.

## IV. CONCLUSIONS

TPS dose calculations based on extended CT were shown to agree with film measurements and provided superior accuracy to standard CT in some cases. In patient plans, the two techniques provided very similar dosimetric results. Together, the observations indicate that extended CT could replace the standard CT method for clinical planning. These observations for extended CT held true over a range of metal types, geometries, and different locations with respect to the metal sample. In some cases, manual contouring resulted in systematic error. While our phantom study used AP/PA beams, arc or multifield plans would likely be affected to a lesser extent in the presence of manually performed contouring or CT number assignment errors.^(14)^

Our results indicate that, for most cases, doing nothing to override HU values actually produces more accurate and consistent results than user‐dependent contouring and overrides. However, it should not be assumed that this is always the case. Depending on the beam arrangement and size of the metal, there are likely to be scenarios using standard CT where a manual override is beneficial. Direct calculation from extended CT eliminates time required for metal contouring, need for physics consultation in manual CT number assignment, and potential for introduction of additional uncertainty by these two subjective, manual steps. Patient plan examples showed generally equivalent agreement between extended CT and standard CT methods, and maximum point discrepancy of less than 1.5%.

To implement the practice of dose calculation based on extended CT scale images requires a new HU‐to‐RED conversion curve containing data points for metals to be stored in the TPS. Once implemented, our study suggests that use of extended scale CT in the presence of metal implants results in accurate, clinically viable dose calculations. The use of extended CT scale for direct dose calculation can potentially reduce systematic uncertainties related to manual contouring of metals and improve workflow efficiency in the treatment planning process.

## COPYRIGHT

This work is licensed under a Creative Commons Attribution 3.0 Unported License.

## Supporting information

Supplementary Material

Supplementary Material
