# Supplementary figures and images for: Treatment planning for metals using an extended CT number scale
Source: J Appl Clin Med Phys. 2016 Nov 8;17(6):179–88. doi: 10.1120/jacmp.v17i6.6153 (PMC5690522; doi:10.1120/jacmp.v17i6.6153)

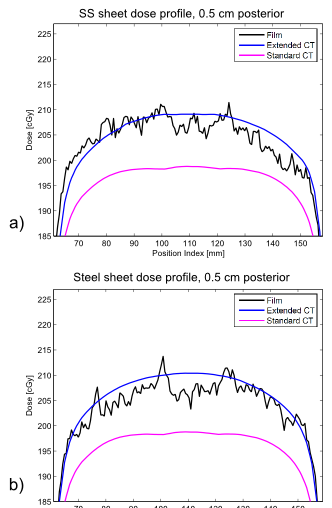

Supplement: Supplementary file 1 — Supplementary Material [file ACM2-17-179-s001.png]

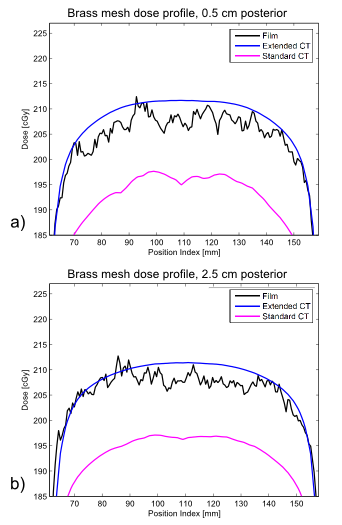

Supplement: Supplementary file 2 — Supplementary Material [file ACM2-17-179-s002.png]
